# Development of the SAFE Checklist Tool for Assessing Site-Level Threats to Child Protection: Use of Delphi Methods and Application to Two Sites in India

**DOI:** 10.1371/journal.pone.0141222

**Published:** 2015-11-05

**Authors:** Theresa S. Betancourt, Stephanie S. Zuilkowski, Arathi Ravichandran, Honora Einhorn, Nikita Arora, Aruna Bhattacharya Chakravarty, Robert T. Brennan

**Affiliations:** 1 Department of Global Health and Population, Harvard T. H. Chan School of Public Health, 665 Huntington Avenue, Boston, MA, 02115, United States of America; 2 Learning Systems Institute and Department of Educational Leadership and Policy Studies, Florida State University, University Center C4600, Tallahassee, FL, 32306, United States of America; 3 FXB Center for Health and Human Rights, Harvard University, 651 Huntington Avenue, Boston, MA, 02115, United States of America; 4 Indian Institute of Public Health–Delhi, Public Health Foundation of India, Plot No. 47, Sector 44, Institutional Area, Gurgaon, 122002, India; Massachusetts General Hospital, UNITED STATES

## Abstract

**Background:**

The child protection community is increasingly focused on developing tools to assess threats to child protection and the basic security needs and rights of children and families living in adverse circumstances. Although tremendous advances have been made to improve measurement of individual child health status or household functioning for use in low-resource settings, little attention has been paid to a more diverse array of settings in which many children in adversity spend time and how context contributes to threats to child protection. The SAFE model posits that insecurity in any of the following fundamental domains threatens security in the others: Safety/freedom from harm; Access to basic physiological needs and healthcare; Family and connection to others; Education and economic security. Site-level tools are needed in order to monitor the conditions that can dramatically undermine or support healthy child growth, development and emotional and behavioral health. From refugee camps and orphanages to schools and housing complexes, site-level threats exist that are not well captured by commonly used measures of child health and well-being or assessments of single households (e.g., SDQ, HOME).

**Methods:**

The present study presents a methodology and the development of a scale for assessing site-level child protection threats in various settings of adversity. A modified Delphi panel process was enhanced with two stages of expert review in core content areas as well as review by experts in instrument development, and field pilot testing.

**Results:**

Field testing in two diverse sites in India—a construction site and a railway station—revealed that the resulting SAFE instrument was sensitive to the differences between the sites from the standpoint of core child protection issues.

## Introduction

Recent years have brought an important shift in child protection approaches toward recognition of the multi-faceted, interrelated, and interdependent nature of children’s security needs within the context of the rights of the child [1]. Of particular importance, a relatively recent emphasis on systems strengthening [2, 3] suggests that efforts must be put in place to improve coordination, cooperation, and integration of the multitude of players working in the child protection field, especially in regions characterized by numerous international NGOs and donor-driven child protection interventions [4, 5]. In order for organizations to share best practices and work effectively in collaboration with each other, rigorous evaluation tools are needed to provide multi-faceted assessments of child protection indicators across various sites in both the short and long term.

Rigorous evaluation tools are needed to provide effective, multifaceted, and comprehensive humanitarian interventions and to build the evidence base for effective site-based tools focused on monitoring and evaluating threats to children. The dearth of programs utilizing site-based tools to evaluate the impact of a program or intervention on children and families living in settings of adversity indicates the need for a greater emphasis on the development of such tools. We reviewed the available literature on assessing threats to child protection in community, home and other settings where children spend time. One measure that is widely used is the HOME (Home Observation for Measurement of the Environment) inventory, which provides a strong assessment of a singular household environment (cleanliness, availability of age appropriate toys, adequate food, clothing) and incorporates interactions with key caregivers. However, like many other assessments of child health and development, the HOME reports on the status of an index child within a single home context. It does not focus on the broader site or settings of the household in a manner that would apply to multiple children at once. Prior to developing the SAFE Checklist, a thorough literature review of environmental impact assessments, or site-based tools, was carried out on ProQuest, PsychInfo, PubMed, Google Scholar, and Web of Knowledge using key words including “measure evaluation,” “measure validation,” “validity of measure,” “measure assessment,” “tool validity,” and “tool evaluation,” as well as “child development,” “child protection,” and “site-based child assessment.” After narrowing down the tools, reviewing only scholarly, peer-reviewed journals, only two tools demonstrated rigorous validity, namely the HOME inventory, and the Environmental Rating Scales. Of these tools, only two conduct assessment with children, and neither measure has been generalized to any setting or population [6–8]. While other measures were reviewed, some of these assessment tools were simply checklists created to adhere the U.S. government requirements (*Environmental Assessment Checklist*, *North Dakota Department of Commerce/DCS*) [9], some were checklists that were patient-focused (*Quick Environmental Health Questionnaire*) [10], some were only recently created and published in 2012 (*WHO Quality Rights Toolkit*) [11], and some were still in the process of building evidence for the tool (*UNICEF’s Child Protection Rapid Assessment Tool)* [12]. Thus, a key gap identified in the literature was the lack of site-level measures of conditions relevant to many children at that site, which could be used for monitoring risks and positive resources at a collective level and for evaluating the impact of site-level interventions.

A site-based assessment, or for that matter, a site-based tool, fills an important gap in the assessment of child protection threats to assist in improved policies and programs. As compared to tools which assess an index child or a single household, a site-based tool enables us to understand interconnected domains of child protection and offers a more comprehensive, holistic assessment of the conditions facing many children in a particular setting. Such tools can help to assess and develop corrective measures at a large scale.

The SAFE Model, a rights-based holistic model for child security, was the theoretical foundation for the development of the tool described in this paper [13–16]. The SAFE model provides a framework for analyzing interconnections and interrelatedness between four core domains of children’s basic security needs and rights: Safety/freedom from harm; Access to basic physiological needs and healthcare; Family and connection to others; Education and economic security [13]. The SAFE Model posits that insecurity in any of these fundamental domains threatens security in the others. When children and families are faced with threats to any of these basic security needs, they respond by adopting survival strategies that can take adaptive forms or risky forms (with cascading negative effects on other dimensions of child security and well-being) [13]. For example, to overcome family economic insecurity, some families may send their child away to work, while others may seek out a small loan program to start a small business. Sending children away to work can have cascading negative effects on children’s safety and family attachment relationships, which can further imperil an already vulnerable child. The SAFE model provides a mechanism to identify adaptive strategies that should be supported rather than supplanted while also illuminating risky strategies in order to inform the development of interventions to effectively address the needs of vulnerable children and families.

The present paper discusses the development and refinement of the SAFE Checklist, which was developed to capture a broad-based set of indicators critical to the SAFE model of children’s security needs and rights. The purpose of the SAFE Checklist is to provide a monitoring tool for use at sites or settings which are not confined to a single household (e.g., a refugee camp, a migrant work site) to improve accountability of governments and NGOs providing services for vulnerable children and families to monitor their basic security needs and rights at a site level in order to inform action. The tool is intended to highlight areas for improvement within existing child protection programs and inform the development of interventions using indicators applicable across a variety of settings. Furthermore, the SAFE checklist is intended to elicit multiple perspectives, including NGO workers, caregivers, and children themselves, in order to uncover strengths and weaknesses traditional studies administered only to individual actors (children or caregivers) or high level officials may miss.

**Fig 1:**
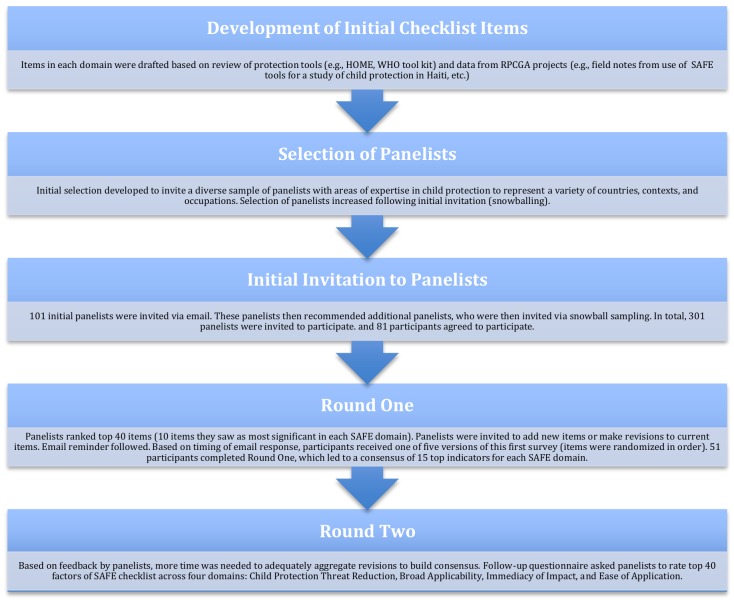
Flowchart of the Delphi panel process

In the following section, we describe our approach to instrument development (see Fig 1), followed by an application of the SAFE Checklist to examine its ability to differentiate between two settings of child vulnerability in India—a construction site in Delhi-NCR (National Capital Region), with a sizable population of migrant laborers and a railway station where street children live in Jaipur. We conclude with a discussion of the strengths and limitations of the Delphi approach and the resulting SAFE Checklist (see S1 File).

## Materials and Methods

### Ethics Statement

All research activities were approved by the Harvard T.H. Chan School of Public Health Institutional Review Board. All materials and responses were obtained with the informed written consent of participants.

### Instrument development process: A modified Delphi approach

The Delphi consensus methodology, which is often utilized in health research and medicine [17–20] is a useful tool for acquiring a consensus of expert opinion in a systematic manner [17, 18, 21]. The Delphi method is a collaboration of a group of experts through the completion of a sequence of surveys combined with rounds of feedback to the expert panel [22, 23]. The Delphi method is especially helpful for bringing together specialists and professionals from a variety of disciplines who may not be able to communicate as a group due to geographical and time-related scheduling constraints and/or costs [24]. Furthermore, the technique prevents effects related to group dynamics, such as more powerful participants dominating group discussions [17]. Other methods of measuring a variety of health priorities for research among children include the Child Health and Nutrition Research Initiative (CHNRI), which has gained support in its ability to reduce personal biases through a unique scoring guide; yet, the technique is not free from bias as the criteria for expert opinion may limit the group size [25–27]. Still, researchers choose to use the Delphi method given its longstanding presence in research methodology, its anonymity, and its ease of utilization [22, 23, 28].

Key components of the Delphi method as applied in our SAFE Checklist development include expert input, anonymity, iteration, controlled feedback, and statistical summary of the collective responses [23]. The different steps taken in our modified Delphi approach are presented in Fig 1. Throughout each round of identifying and evaluating the topic, panelists can communicate their reasoning, while also re-assessing their decisions based on other panelists’ explanations. This is done through controlled, anonymous feedback, whereby the researcher team forwards the collective opinions back to the panelists with each survey round [29, 30]. The Delphi method is particularly beneficial to theory development because it fosters extensive identification and understanding of factors of interest and the generalizability of the resulting constructs [31]. The Delphi approach is also seen as a well-matched system for contributing perspectives in new or emerging fields, where ideas may be inconsistent or controversial [21]. Furthermore, this method allows members of the group to share terms and language that can help to harmonize or clarify communication [32].

We utilized a ‘modified’ Delphi method [33, 34] in order to accommodate the needs of our field partners piloting the first round of the SAFE Checklist in India. Under the modified approach, rather than beginning the process with open-ended questions, we restricted the ability of the panelists to respond to the research question by developing an inclusive version of the Delphi questionnaire, wherein an initial list of items were provided in a structured format, before Round 1 of the exercise. Given this accelerated starting point, we predetermined that we would only conduct two rounds of review by the panel due to the heavy commitment of the panel members and the logistical considerations of maintaining communications with such a diverse international panel.

Round 1 of the SAFE Delphi exercise called upon 51 child protection experts working on issues relating to the health and well-being of children and families from North & South America (23), Europe (13), Australia (2), Asia (7), Africa (3), the Middle East (1), and Africa/Europe (2). Of these, 18 were from international NGOs, 16 were from research/advocacy groups, 8 were from grassroots NGOs, and 9 were either independent child protection consultants or worked for organizations that were some combination of international NGO, research/advocacy group, and grassroots NGO. A total of 26 experts were women, and 25 were men (see Fig 1).

The initial items included in the Delphi questionnaire in Round 1 were based on the four core domains of the SAFE Model of child protection with items formulated to capture the SAFE domains (e.g., “Majority of children have identified attachment figure/caregiver”) and additional items drawn from literature reviews of related child status monitoring tools and other measures, (e.g., “Significant proportion of children exhibit signs of physical abuse [frequent injuries, unexplained bruises, welts, cuts"]) [13–16]. Therefore, in addition to key indicators extrapolated from case studies and fieldwork at the Research Program on Children and Global Adversity (RPCGA), this literature review was done using the search terms “child protection tools,” “child status monitoring,” “child protection assessments,” and “child status assessments,” which revealed a set of five measures (snowball sampling with Delphi experts revealed no additional measures to be considered). As a result, 115 items/indicators (25 items regarding Safety/freedom from harm; 30 items regarding Access to basic physiological needs and healthcare; 22 items regarding Family and connection to others; and 38 items regarding Education and economic security) were developed in the first survey used for the first round in the Delphi method.

To mitigate limitations of beginning the Delphi process with a pre-determined set of domains and sample questions, we requested the feedback and brainstorming of the expert panel in an open-ended form [34]. The Delphi panelists were asked to select key indicators of inclusion for the SAFE item pool from a pre-determined list of indicators; however, they were also encouraged to provide feedback and revisions to the list by adding, editing, or commenting on items. Panelists were asked to choose the ten most important indicators of child health and well-being per each of the SAFE domains from the list of child security issues mentioned above. Responses were collated resulting in a distilled list of indicators determining key items of evaluation within each of the four SAFE domains. Participants were given the opportunity to offer new items as well as to offer feedback about pre-selected items drawn from the literature and theory, and about the process. Table 1 shows the results of this exercise.

**Table 1:** SAFE items in rank order of most often chosen (from highest to lowest) of ten most frequently chosen by domain, first round.

**Safety from harm**
1. Significant proportion of children exhibit signs of sexual abuse (emotional consequences/fear, sexualized behavior, STDs and/or pregnancy under the age of 14)
2. Significant proportion of children exhibit signs of physical abuse (frequent injuries, unexplained bruises, welts, cuts)
3. Significant proportion of children exhibit signs of emotional abuse (excessively withdrawn, fearful, anxious)
4. Children are involved in armed conflict (active participants in war, regional violence, and/or gang violence)
5. Access to trusted individuals/organizations that can provide protection (security guards, police)
6. Government issued child protection policy is monitored and enforced at site
7. Significant proportion of children exhibit signs of neglect (filthy clothes, unbathed, playing in unsafe environments)
8. Site has safe, supervised spaces reserved for children which are non-toxic and free from hazards (crèches, play areas)
9. Majority of children are exposed to stagnant and/or unclean water
10. Grievances are effectively monitored and addressed at site
**Access to health care and basic physiological needs**
1. Access to clean drinking water
2. Access to nutritious food
3. Access to health care facilities (hospitals, community health centers, rural clinics)
4. Majority of children are immunized
5. Access to reproductive health services (pre-natal care, post-natal care)
6. Access to safe, dry shelter
7. Access to health care personnel (doctors, nurses, volunteers, community health workers, community healers)
8. Access to adequate quantity of food
9. Access to basic health services even without proper legal identification
10. Access to clean, gender separate latrine and bathing facilities
**Family and connection to others**
1. Majority of children have identified attachment figure/caregiver
2. Children are unaccompanied, orphaned, and/or displaced at site
3. Incidence of domestic violence
4. Orphaned and/or abandoned children receive social care (orphanages, foster care homes)
5. Children have proper legal documentation (birth certificate)
6. Access to tracing and unification system (if necessary)
7. Incidence of community violence (assault, burglary, use of weapons, muggings, sound of gun shots, gang violence)
8. Incidence of discrimination due to race, gender, sex, illness, and/or caste
9. Access to social service/child welfare organization
10. Access to shelter and/or health care provider for victims of child abuse
**Education and economic security**
1. Families can afford basic needs (food, shelter, water, clothing)
2. Access to free primary school education
3. Children can safely travel to school
4. Access to social welfare assistance for vulnerable children and families
5. Access to vocational training opportunities for older children and adults
6. Access to early education learning program (pre-school, kindergarten)
7. Incidence of child labor
8. Majority of girls complete secondary school
9. Majority of girls complete primary school
10. School employs good quality teachers

The open-ended feedback from the Delphi exercise pointed to the need to significantly reword and edit the items. Many panelists objected to the “majority of…” construction in some items. Others felt that some items were redundant in that some closely related child protection topics were spread across multiple items that could be combined. Several respondents commented that selecting the ten most important items was difficult without knowing the particular type of site and/or geographical location, as the relevance of some issues would vary depending on these issues.

These concerns were significant enough that several steps were taken to revise the items prior to a second round of evaluation. First, significant internal resources were focused on finding an item format and wording that would work with the majority of items that would address the concerns of panelists who criticized the wording. Second, the cumulative rating of items was utilized whereby items most frequently included in the ten most important lists were ranked highest and items least frequently marked were ranked lowest. Experts working for survey research firms reviewed all items for technical quality. The primary aim of this review and the targeted interviews was to ensure that within each SAFE domain the final questionnaire would have adequate content validity; that is to say that for each of the domains there was adequate coverage of the topic and nothing important was omitted.

For items referring to the status of children at a site, the revised common item format asked the respondents to assess “how many of the children (ages 0–18) at the site” fit the description in the item, and to do so to the best of their knowledge, which would help to encourage respondents with imperfect knowledge to respond. The viability of the item format and response format were reviewed with our India field team where the first version of the resulting SAFE Checklist would be pilot tested.

In Round 2 all of the original participants were re-contacted and provided with a synopsis of the results from Round 1. Round 2 of the SAFE Delphi exercise included 17 of the original 51 child protection experts who participated in Round 1. These experts were from North America, Central America, Europe, Australia, and Asia working in research institutions, universities, grassroots organizations, international NGOs, and human rights based advocacy groups on issues relating to the health and well-being of children and families. Ten of the SAFE Delphi experts who participated in Round 2 were women and seven were men. In this round, the panelists were asked to rank order and choose the five most important items to be used in the SAFE Checklist that corresponded to each of the four different SAFE domains. The ranked items of the SAFE Checklist from Round 2 are shown in Table 2. The rationale for having participants rank the five top items was to ensure that the most important items in each domain were retained, but to reduce burden on participants who indicated that ranking 10 items per each of the 4 domains had been burdensome in Round 1. Round 2 was seen as clarifying and refining round to arrive at a scale of manageable length, strong applicability and comprehensiveness.

**Table 2:** SAFE items in rank order (from highest to lowest) of highest ranked by domain, second round.

**Safety from harm**
To the best of your knowledge how many of the children (ages 0–18) at the site have been physically abused at the site (burning, hitting, shaking, kicking, beating)?
To the best of your knowledge how many of the children (ages 0–18) at the site have been physically sexually abused at site (sexual exploitation, sodomy, rape, incest, intercourse, genital touching)
To the best of your knowledge how many of the children (ages 0–18) at the site have been threatened, intimidated, yelled at harshly, name-called, accused, and/or humiliated at the site?
To the best of your knowledge how many of the children (ages 0–18) have been living without a parent or caregiver at the site?
To the best of your knowledge how many of the children (ages 0–18) have been living in a space that is not protected from cold, damp, heat, rain, and/or wind?
To the best of your knowledge, how many young people (ages 0–18) were brought to the site via exploitation and/or trafficking?
To the best of your knowledge how many of the children (ages 0–18) have access to police whom they trust to provide protection?
To the best of your knowledge how many of the children (ages 0–18) at the site have friends/or peers whom they trust to provide protection?
To the best of your knowledge how many of the children (ages 0–18) currently have access to separate washing facilities for girls and boys?
To the best of your knowledge how many of the children (ages 0–18) have been killed by violence at the site?
**Access to health care and basic physiological needs**
To the best of your knowledge how many of the children (ages 0–18) at the site have access to clean drinking water?
To the best of your knowledge how many of the children (ages 0–18) at the site have access to free health services?
To the best of your knowledge how many of the children (ages 0–18) at the site have access to health services that are easy to travel and from site?
To the best of your knowledge how many of the children (ages 0–18) at the site have access to sexual health services such as testing, counseling, and treatment for HIV and/or other sexually transmitted infections?
To the best of your knowledge how many of the children (ages 0–18) at the site have access to pregnancy services such as prenatal care and/or post-natal care?
To the best of your knowledge how many of the children (ages 0–18) at the site have enough to eat?
To the best of your knowledge how many of the children (ages 0–18) at the site need medical care but do not receive it because they cannot afford it?
To the best of your knowledge how many of the children (ages 0–18) at the site have received routine immunizations?
To the best of your knowledge how many of the children (ages 0–18) at the site have access to resources such as condoms and/or birth control pills?
To the best of your knowledge how many of the children (ages 0–18) at the site have access to doctors, nurses, and/or community health workers?
**Family/connection to others**
To the best of your knowledge how many of the children (ages 0–18) at the site experience physical violence such as burning, hitting, punching, shaking, kicking, and/or beating IN THE HOME?
To the best of your knowledge how many of the children (ages 0–18) at the site experienced emotional violence such as humiliation, verbal assaults, name-calling, accusing, threatening, and/or intimidation by a parent or family member, and/or caregiver IN THE HOME?
Violence is pervasive and common at the community in which the site is located.
To the best of your knowledge how many of the children (ages 0–18) at the site have a relationship with a trusted individual who provides them with care?
To the best of your knowledge how many of the children (ages 0–18) at the site have access to a social Service and/or child welfare organization
To the best of your knowledge how many of the children (ages 0–18) at the site have access to services that reunite separated children to their families?
To the best of your knowledge how many of the children (ages 0–18) at the site are discriminated against due to race, ethnicity, gender, caste, illness, disability, and/or religion IN THE HOME?
To the best of your knowledge how many of the children (ages 0–18) at the site have access to at least one family member who abuses alcohol?
To the best of your knowledge how many of the children (ages 0–18) at the site have to care for younger children without the help of an adult?
To the best of your knowledge how many young girls (ages 0–18) at the site are married?
**Education/economic security**
To the best of your knowledge, how many of the children (ages 0–18) and/or families have access to social/welfare assistance
To the best of your knowledge, how many of the children (ages 0–18 and/or families have access to micro-lending schemes from banks?
To the best of your knowledge, how many of the children (ages 0–18 and/or families are unable to afford basic needs (food, water, shelter, clothing)?
To the best of your knowledge, how many of the children (ages 0–18 at the site attend school?
To the best of your knowledge, how many children (ages 0–18 at the site are emotionally abused or bullied AT SCHOOL (fear, humiliation, verbal assaults, name-calling, accusing, threatening, and or intimidation) by students, teachers, and/or other people?
To the best of your knowledge, how many children (ages 0–18) at the site are physically abused by students, teachers, and/or other people AT SCHOOL (burning, hitting, punching, shaking, kicking, and/or beating)?
How many of the children (ages 0–18) and/or families owe money to others?
How many of the children (ages 0–18) and/or families have access to vocational training opportunities?
How many of the children (ages 0–18) are sexually abused at school (sexual exploitation, sodomy, rape, incest, intercourse, genital touching) by students, teachers, and/or other people?
How many of the children (ages 0–18) are on the street, at the market, at the railways, and/or at the worksite during school hours?

In Round 2 of the Delphi exercise, the response scale was broken into five percentage categories (0%, 1–25%, 26%–50%, 51%–75%, 76%–100%) without verbal anchors. Finally, the draft items were circulated to three outside experts on survey research, including one with low- and middle-income country (LMIC) experience, including India, for technical quality.

After Round 2, the written form of the items were sent to three external experts on questionnaire construction, including one with experience in India, for review of wording and format. The instrument was forward translated and independently back translated from English to Hindi by a team of professional translators regularly used by the Public Health Foundation of India (PHFI). After translation, the instrument was sent for review by the team of four Indian research assistants who had extensive experience working as either teachers and/or counselors with the study population. The instrument was then revised based on their recommendations, and then pre-piloted with a small group of six respondents (3 adolescent girls between the ages of 12–15, and three adolescent boys between the ages of 12–15), recruited from youth living with their families at a construction site encampment. These respondents were immediately debriefed using techniques of cognitive testing [35] about their responses on a question-by-question basis regarding the clarity of the items. The cognitive testing participants were also asked about the adequacy of the response categories for the respondents to accurately express their knowledge and understanding for each item [35]. Revisions to the instrument resulting from the pre-piloting experience were conducted in consultation with the India field staff and were undertaken in the English master version.

### Instrument functioning: a brief comparison of two sites

Following the development of the SAFE Checklist via Delphi methodology, the team piloted the tool in two sites in India: a Delhi-NCR (National Capital Region) construction site where migrant workers had brought their families, including young children, while they were engaged in temporary work, and a setting where children were living on streets surrounding the railway station in Jaipur.

#### Study sites

The Delhi-NCR site was chosen to represent the living and working conditions faced by India’s vast migrant population. It is estimated that 40 million Indians work as migrant laborers in the construction and infrastructure industries alone [36]. Children commonly accompany their migrant parents, living at construction sites. Laborers and their children face a number of risks in such a lifestyle. Parents work long days for low wages [37–39] and live in substandard housing [37, 38, 40, 41] with poor sanitation, and little access to clean water [38, 39, 41]. Researchers have found that migrants are at increased risk of experiencing sexual violence [39, 42]. Families may not have access to consistent schooling or other social services, due to their frequent moves. In other countries, mobility has been shown to lead to increased risk of child maltreatment as well as diminished social capital and support [43–46]. In sum, migrant families working in urban areas experience poor quality of life in exchange for employment.

A non-governmental organization, Mobile Crèches, assisted in the selection of the construction company and site for the piloting of this instrument in Delhi-NCR. Although the company was interested in corporate social responsibility and welcomed the research project, the site was, in fact, quite dangerous for workers and their families. Workplace accidents were common, due both to lax adherence to regulations and language barriers between migrant laborers and company managers. In addition, there was poor access to emergency care facilities in close proximity to the construction site, which made it difficult to provide timely medical support after worksite-related accidents. Workers were often exhausted, due to their work schedule, poor housing, lack of weather-appropriate clothing, and mosquitoes; which interfered with sleep. A child care center was available until 5 pm, but parents generally worked until 8 pm, leaving children unsupervised for at least three hours per day. Older children, especially girls, were often left alone at home which could have been risky in itself but also exposed them to hazards while cooking and performing other household chores unmonitored. Certain sections of the labor settlements were still using traditional cook stoves, subjecting women and children to the ill effects of indoor air pollution. There were no safe places for children to play outside of the center. At night, the housing area was poorly lit, making it dangerous for women and children to use the toilets or leave their homes for any reason. While children were living with employed parents and were therefore in some ways fortunate, they were living in an environment that posed risks to child health and safety.

In Jaipur, the state capital of Rajasthan, we selected one railway site as representative of the living conditions of children living on the streets. India has the largest number of street children in the world [47]. While it is difficult to obtain estimates of this mobile and marginalized population, one government report states that there are 11 million children living on the streets in India’s cities [48]. Street children are not only at risk of hunger, exposure, and illness, but also of exploitation and abuse. In Rajasthan, almost all street children reported experiencing moderate or severe abuse [49].

The children living at this railway station were largely living apart from their families and worked to support themselves, usually by rag-picking and doing odd jobs such as shoe polishing and vending inexpensive food items. Some families would spend time nearby the tracks and send their young children to beg. As found in other parts of India, most street children were addicted to inhalant drugs—often whitener/solution—and this resulted in behavior that escalated violent conflicts with other children, adults, and the Railway Police Force (RPF). Abuse of these kinds of drugs can lead to a range of serious health problems, including lung, stomach, and heart ailments [50]. In some cases, while in a state of intoxication, children were known to engage in cutting using a blade or knife to carve into their hands and legs [51, 52]. Police brutality and exploitation of street children were described as common. After falsely incarcerating children, the railway police were known to accept bribes from their family members to allow their release. Children living in and around the Jaipur railway station were generally not attending school, due to their need to work as well as lack of identification and documentation of previous attendance. Bullying, sexual exploitation, and physical abuse from peers, the railway mafia, and the police were the norm; girls and all children under 16 were particularly at risk. Disabled children, especially those with mental illnesses, were often abandoned by their parents and left unsupported by non-governmental organization (NGO) workers. A few small NGOs were in operation providing medical and feeding programs and some educational and vocational training activities to try and draw children out of the dangerous survival strategy [14] of living in an around the Jaipur station engaged in begging or rag picking.

#### Data Analysis

All SAFE checklist items were ordinal in nature and the sample sizes were small. In order to best accommodate these data, an exact version of Mann-Whitney of the Mann-Whitney U test as implemented in SYSTAT 13 [53, 54] was used to test for differences between the Jaipur and Delhi-NCR sites as assessed by adult caregivers at the two sites.

## Results

**Fig 2:**
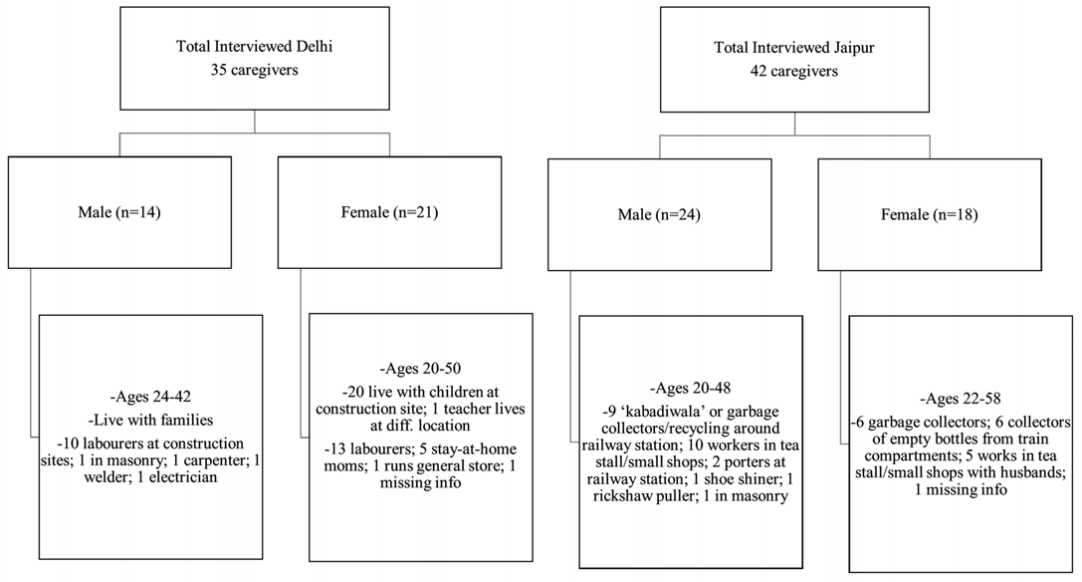
Adult caregivers interviewed at Jaipur and Delhi-NCR sites

Across multiple reporter groups, children and adult caregivers, both male and female, differences between the Jaipur and Delhi-NCR sites were detected on almost all items. (Given concerns about low literacy for use of self-report questionnaires, all checklist questions for both sites were administered orally by trained local interviewers with training and experience in working with vulnerable children. The mode of data collection was in-person interviews and Focus Group Discussions.) In the table below we chose to display comparisons of five selected items for the adult caregiver samples. Many of the children in Jaipur were intoxicated due to use of inhalants, so we elected not to report comparisons between the sites based on child reports. Adult NGO workers (*N* = 10) were surveyed only in Delhi-NCR, so no cross-site comparisons were possible for that sub-group. A breakdown of the adult caregivers interviewed at the two sites is shown in Fig 2.

An examination of responses to representative items across the two sites as shown in Table 3, demonstrates the ability of the tool to identify disparate environments. Children at the Delhi-NCR site, whose parents were employed, were markedly better off than children at the Jaipur site, who were largely fending for themselves. The median response for having enough to eat, as well as for school attendance, was 5 in Delhi-NCR, indicating that most children had access to food and schooling. As would be expected, given that these families generally have access to company-provided on-site housing, the median response for the item regarding housing was 1, indicating that few children in Delhi-NCR lived in such conditions. Similarly, few of the Delhi-NCR children “need to earn money for the household,” and few children used drugs. In contrast, in Jaipur the median response to the item asking respondents: “To the best of your knowledge, how many of the children… currently: use drugs or other substances such as whitener, Iodex, puncture fluid, smack, and/or solvent?” was a 4, which expresses the belief that most children were using drugs.

**Table 3:** Comparison of Jaipur and Delhi-NCR on selected SAFE items.

SAFE item^1^	Median Jaipur (N)	Median Delhi (N)	Mean^2^ Jaipur	Mean^2^ Delhi	Mann-Whitney U	*P-*value^3^
Children use drugs or other substances	4 (51)	1 (43)	4.23	1.16	25.0	< .001
Children have enough to eat	3 (50)	5 (43)	3.36	4.67	1875.5	< .001
Children live in a space unprotected from environment	4 (50)	1 (41)	3.62	2.00	374.0	< .001
Children attend school	1 (35)	5 (40)	1.63	4.45	2167.0	< .001
Children need to earn money for the household	5 (36)	1 (41)	4.17	1.22	37.0	< .001

^1^Response scale: 1 = None (0%), 2 = Few (1%-25%), 3 = Some (26–50%), 4 = Most (51%-75%), 5 = Almost all/All (76%-100%).

^2^Means of ordinal scales are provided only to assist in comparing Jaipur ratings to Delhi ratings.

^3^P-value for exact Mann-Whitney U.

Most Jaipur site children were characterized as living in spaces unprotected from the environment (median 4), and almost all had to earn money for their households (median 5). Few children were attending school (median 1).

These ratings accurately reflect the observable differences in the sites. The Jaipur site provides little protection of any kind, and children fill their days in opportunistic earning precluding any chance of education or economic stability. As the risk of theft is high, especially during nights when they are asleep, children tend to spend their money before the end of the day, often using it to buy whitener or other inhalant drugs. There are significant risks to the healthy development of children at the Jaipur site that exceed those in Delhi-NCR. A future publication will present a complete comparison between the two pilot sites, providing an example of how the tool is able to differentiate between child needs in disparate settings and the value of multiple perspectives and multiple informants in assessing a site.

## Discussion

The purpose of using the Delphi method was to generate expert collaboration and consensus regarding the conceptualization and measurement of child protection and security for the formulation of the SAFE checklist. As a result of our first round of Delphi feedback, we undertook significant revision in the construction of individual items and in the streamlining and refining of the content of the SAFE checklist. Both changes were substantial enough that outside expertise was brought in before circulating a revised version of the checklist to Delphi panelists in the Round 2 Delphi exercise. Although we have endeavored to create a checklist focusing on core concerns of child protection and welfare that crosses many boundaries, concerns raised by panelists that site type and setting may determine priorities are not lost on us. For example, in sites with high rates of trafficking and child prostitution, protection against and treatment for STDs and HIV and personal safety as well as the other related hazards may be central concerns; in sub-Saharan Africa dealing with HIV-infected parents/caregivers may be relevant; while in other locations dealing with war trauma and separation from family might be central. In some cases, such as Indian railway sites, schooling may be so far from the experiences of most children that questions about the provisions at school are irrelevant to their experiences. Thus, while we believe that there are universal core issues within the SAFE framework, we also believe that there are site-specific concerns that might be added to the questionnaire in a modified, module-specific format, while other aspects of the questionnaire might not be probed in certain sites, if the area(s) probed is/are largely irrelevant. So, for example, there might be additional modules that can be added to a core SAFE questionnaire to deal with site-specific issues including trafficking/prostitution, involvement of youth in conflict, impact of HIV on families, tropical diseases, and quality of schooling.

Following the Delphi exercise, our pilot research in India illuminated the strengths and weaknesses of the SAFE Checklist, particularly the effectiveness of said site-based measure in real world settings. Further work with field research staff has demonstrated that for some respondents, the use of percentages without verbal anchors is not useful for many categories of respondents. Accordingly, our response scale has been further revised since the Delphi review to add verbal anchors [None (0%), Few (1%-25%), Some (26%-50%), Most (51%-75%), Almost all/All (76%-100%)]. Visual analogues for this scale are also available for low-literate populations and can be tested in other settings. In general, however, the piloted version accomplished the goal of identifying distinct risk and protective factors in two settings in urban India.

This collaborative tool development approach, as well as the SAFE Checklist itself, are promising models for program planners, managers, and researchers working in the fields of child development and protection in low-resource settings. In settings where existing, western instruments are not appropriate or do not capture relevant issues, rigorous methods must be used to create new ones. Data is only as reliable as the tools used to collect it, and the time needed to conduct the Delphi approach and the further steps toward refinement we employed is therefore well spent. The findings that result from the usage of the SAFE Checklist may be used to identify problem areas or strengths that may be leveraged, and is quick and inexpensive to administer. Future applications of the SAFE Checklist in various countries will allow for comparative studies of its effectiveness as a holistic measure of children’s environments.

### Limitations

Though the Delphi method can help to overcome weaknesses of in-person focus groups or other group consultations such as pressure or influence from dominant personalities, group dynamics relating to becoming too focused on certain domains or too broad in areas of focus, becoming side-tracked, and/or losing sight of the initial goal [23, 31], this method is not without its flaws. Not only does this method require additional effort and time to complete, but retention of Delphi panelists given deadlines to return comments [23] can be a challenge and certainly affected our participant numbers between Stage 1 and Stage 2. Other important limitations of the Delphi approach include poorly designed and executed surveys, as well as inappropriate choice of panelists and selection bias [21, 23]. In our case, the use of highly committed and widely dispersed experts, many with only weak associations with the investigators, if any, meant a dramatic drop off in response rates between the first and second rounds and that efforts at a third round using the original panelist would have been futile. To compensate for these limitations, we relied upon both internal and external experts in instrument construction because a great deal of commentary in Round 1 concerned the specifics of wording of the proposed survey items rather than content. We also sought the input of experts in content in each of the SAFE domains to augment the information obtained from the first round of evaluation by the panel.

## Conclusion

The SAFE Checklist, developed via a modified Delphi process, is the first holistic child welfare and protection assessment for children living under adverse circumstances applicable at a site level. None of our panelists questioned the utility of such an approach. Our modified two-round Delphi approach was enhanced with substantial expert input and has yielded a tool reflecting the priorities and thinking of top experts engaged in a variety of roles in the child protection and children and adversity fields. Informed feedback suggested that while there are definite core priorities, there is a need to address additional site-specific priorities that might be added on in a modular approach. The tool was also seen as having high utility and contributing to a measurement gap that requires moving beyond assessments of individual children or individual households to promote child well-being in situations of adversity.

## Supporting Information

S1 File SAFE Checklist. (PDF)
