# Supplementary material for: Development of the SAFE Checklist Tool for Assessing Site-Level Threats to Child Protection: Use of Delphi Methods and Application to Two Sites in India
Source: PLoS One. 2015 Nov 5;10(11):e0141222. doi: 10.1371/journal.pone.0141222 (PMC4634981; doi:10.1371/journal.pone.0141222)
Supplement: S1 File — (PDF) [file pone.0141222.s001.pdf]

SAFE Checklist

To Advance the Protection of Children & Youth

PART 1

*This set of questions asks about things relating to the health of children (ages 0-18) at the [NAME OF SITE]. **Please circle the answer to the following questions to the best of your knowledge.** You may not know all the answers. If you cannot answer a question, please leave the question blank.*

	To the best of your knowledge, how many of the children (ages 0-18) at the [NAME OF SITE] currently have access to:	None (0%)	Few (1%-25%)	Some (26%-50%)	Most (51%-75%)	Almost all/All (76%-100%)
1.1	Clean drinking water?	None	Few	Some	Most	Almost all/All
1.2	Hospitals and/or health clinics?	None	Few	Some	Most	Almost all/All
1.3	Doctors, nurses, community health workers, and/or health volunteers?	None	Few	Some	Most	Almost all/All
1.4	Free health services?	None	Few	Some	Most	Almost all/All
1.5	Health services that can be reached by accessible roads?	None	Few	Some	Most	Almost all/All
1.6	Health services that can be reached by public transportation, such as bus or auto rickshaw?[a]	None	Few	Some	Most	Almost all/All
1.7	Health services that are open 24 hours a day and 7 days per week?	None	Few	Some	Most	Almost all/All
1.8	Sexual health services such as testing, counseling, and treatment for HIV and/or other sexually transmitted infections?	None	Few	Some	Most	Almost all/All
1.9	Medical treatment for diarrhea?	None	Few	Some	Most	Almost all/All
1.10	Medical treatment for dengue?	None	Few	Some	Most	Almost all/All
1.11	Medical treatment for malaria?	None	Few	Some	Most	Almost all/All
1.12	Medical treatment for coughs and/or difficulty breathing?	None	Few	Some	Most	Almost all/All

[a] Item adapted for use in India.

Do not distribute without written consent. For more information, please contact:

Theresa S. Betancourt, Sc.D., M.A.
Director, Research Program on Children and Global Adversity | Harvard T.H. Chan School of Public Health
Department of Global Health and Population | Email: Theresa_Betancourt@harvard.edu

SAFE Checklist

To Advance the Protection of Children & Youth

	**To the best of your knowledge, <u>how many of the girls and/or women</u> at the [NAME OF SITE] currently <u>have access to</u>:**	**None (0%)**	**Few (1%-25%)**	**Some (26%-50%)**	**Most (51%-75%)**	**Almost all/All (76%-100%)**
1.13	Medical care before having a baby and/or medical care after having a baby?	None	Few	Some	Most	Almost all/All
1.14	Birth control methods such as condoms and/or birth control pills?	None	Few	Some	Most	Almost all/All
1.15	Abortion services—having trained medical workers end a pregnancy?	None	Few	Some	Most	Almost all/All

	**To the best of your knowledge, <u>how many of the children</u> (ages 0-18) at the [NAME OF SITE] currently:**	**None (0%)**	**Few (1%-25%)**	**Some (26%-50%)**	**Most (51%-75%)**	**Almost all/All (76%-100%)**
1.16	Use drugs or other substances such as whitener, iodex, puncture tube fluid, smack, and/or solvent?[a]	None	Few	Some	Most	Almost all/All
1.17	Smoke cigarettes?	None	Few	Some	Most	Almost all/All
1.18	Use alcohol?	None	Few	Some	Most	Almost all/All
1.19	Have enough to eat?	None	Few	Some	Most	Almost all/All
1.20	Eat discarded and/or spoiled food?	None	Few	Some	Most	Almost all/All
1.21	Have problems sleeping?	None	Few	Some	Most	Almost all/All
1.22	Feel tired throughout the day?	None	Few	Some	Most	Almost all/All
1.23	Have received complete routine immunization?	None	Few	Some	Most	Almost all/All
1.24	Need *medical care* but do not receive it because they cannot afford it?	None	Few	Some	Most	Almost all/All
1.25	Need *medication* but do not receive it because they cannot afford it?	None	Few	Some	Most	Almost all/All
1.26	Have died from health-related problems?	None	Few	Some	Most	Almost all/All

[a] Item adapted for use in India.

Do not distribute without written consent. For more information, please contact:
Theresa S. Betancourt, Sc.D., M.A.
Director, Research Program on Children and Global Adversity | Harvard T.H. Chan School of Public Health
Department of Global Health and Population | Email: Theresa_Betancourt@harvard.edu

SAFE Checklist

To Advance the Protection of Children & Youth

PART 2

The next set of questions asks about things relating to children (ages 0-18) and their relationships with their family, friends, and community. ***Please circle the answer to the following questions to the best of your knowledge.*** *You may not know all the answers. If you cannot answer a question, please leave the question blank.*

	**To the best of your knowledge, how many of the children (ages 0-18) at the [NAME OF SITE] currently:**	**None (0%)**	**Few (1%-25%)**	**Some (26%-50%)**	**Most (51%-75%)**	**Almost all/All (76%-100%)**
2.1	Have to care for younger children without the help of an adult?	None	Few	Some	Most	Almost all/All
2.2	Are burned, hit, punched, shaken, kicked, and/or beaten in the home?	None	Few	Some	Most	Almost all/All
2.3	Are threatened, intimidated, yelled at harshly, name-called, accused, and/or humiliated in the home?	None	Few	Some	Most	Almost all/All
2.4	Are inappropriately touched in the home?	None	Few	Some	Most	Almost all/All
2.5	Are raped in the home?	None	Few	Some	Most	Almost all/All
2.6	Witness community violence such as assault, use of weapons, muggings, gang violence, and/or police brutality?	None	Few	Some	Most	Almost all/All
2.7	Are treated badly by others because of race, ethnicity, gender, caste, illness, disability, and/or religion?	None	Few	Some	Most	Almost all/All
2.8	Have at least one family or household member who abuses drugs?	None	Few	Some	Most	Almost all/All
2.9	Have at least one family of household member who abuses alcohol?	None	Few	Some	Most	Almost all/All
2.10	Have a relationship with a trusted individual who provides them with care?	None	Few	Some	Most	Almost all/All
	**To the best of your knowledge, how many of the children (ages 0-18) at the [NAME OF SITE] currently have access to:**	**None (0%)**	**Few (1%-25%)**	**Some (26%-50%)**	**Most (51%-75%)**	**Almost all/All (76%-100%)**
2.11	Social service and/or child welfare organizations?	None	Few	Some	Most	Almost all/All
2.12	Religious, spiritual, dance, arts, and/or sports programs?	None	Few	Some	Most	Almost all/All
2.13	Services that reunite separated children to their families?	None	Few	Some	Most	Almost all/All

[a] Item adapted for use in India.

Do not distribute without written consent. For more information, please contact:
Theresa S. Betancourt, Sc.D., M.A.
Director, Research Program on Children and Global Adversity | Harvard T.H. Chan School of Public Health
Department of Global Health and Population | Email: Theresa_Betancourt@harvard.edu

	**To the best of your knowledge:**	**None (0%)**	**Few (1%-25%)**	**Some (26%-50%)**	**Most (51%-75%)**	**Almost all/All (76%-100%)**
2.14	How many of the young girls (ages 0-18) at the site are married?	None	Few	Some	Most	Almost all/All
2.15	How many of the young boys (ages 0-18) at the site are married?	None	Few	Some	Most	Almost all/All

PART 3

The next set of questions asks about things relating to children (ages 0-18) and their exposure to harm, violence, and physical and emotional safety. ***Please circle the answer to the following questions to the best of your knowledge.*** *You may not know all the answers. If you cannot answer a question, please leave the question blank.*

	**To the best of your knowledge, in the past 6 months, how many of the children (ages 0-18) at the [NAME OF SITE] were:**	**None (0%)**	**Few (1%-25%)**	**Some (26%-50%)**	**Most (51%-75%)**	**Almost all/All (76%-100%)**
3.1	Burned, hit, punched, shaken, kicked, or beaten at the site?	None	Few	Some	Most	Almost all/All
3.2	Inappropriately touched at the site?	None	Few	Some	Most	Almost all/All
3.3	Raped at the site?	None	Few	Some	Most	Almost all/All
3.4	Threatened, intimidated, yelled at harshly, name-called, accused, and/or humiliated at the site?	None	Few	Some	Most	Almost all/All
3.5	Killed by violence at the site?	None	Few	Some	Most	Almost all/All
3.6	Killed by accidents at the site?	None	Few	Some	Most	Almost all/All
3.7	Using weapons such as guns, knives, and/or sharp objects to hurt others at the site?	None	Few	Some	Most	Almost all/All

[a] Item adapted for use in India.

Do not distribute without written consent. For more information, please contact:
Theresa S. Betancourt, Sc.D., M.A.
Director, Research Program on Children and Global Adversity | Harvard T.H. Chan School of Public Health
Department of Global Health and Population | Email: Theresa_Betancourt@harvard.edu

3.8	Living without a parent or another adult who takes care of them at the site?	None	Few	Some	Most	Almost all/All
3.9	Exposed to unsafe chemicals in the air, water, and/or home environment at the site?	None	Few	Some	Most	Almost all/All
3.10	Living in a space that is not protected from cold, damp, heat, rain, and/or wind?	None	Few	Some	Most	Almost all/All
3.11	Left their homes to make money for their families?	None	Few	Some	Most	Almost all/all
3.12	Left their homes due to violence and/or abuse?	None	Few	Some	Most	Almost all/All
	**To the best of your knowledge, how many of the children (ages 0-18) at the [NAME OF SITE] currently have access to:**					
3.13	Toilets?	None	Few	Some	Most	Almost all/All
3.14	Separate toilets for girls and boys?	None	Few	Some	Most	Almost all/All
3.15	Washing facilities?	None	Few	Some	Most	Almost all/All
3.16	Separate washing facilities for girls and boys?	None	Few	Some	Most	Almost all/All
	**To the best of your knowledge, please answer "yes" or "no" to the following questions:**					
3.17	Are there are toilets with locks inside the door?	Yes	No			
3.18	Are there are washing facilities with locks inside the door?	Yes	No			
	**To the best of your knowledge, how many of the children (age 0-18) at the [NAME OF SITE] have access to:**	**None (0%)**	**Few (1%-25%)**	**Some (26%-50%)**	**Most (51%-75%)**	**Almost all/All (76%-100%)**
3.19	Police whom they trust to provide protection?	None	Few	Some	Most	Almost all/All
3.20	Friends and/or peers whom they trust to provide protection?	None	Few	Some	Most	Almost all/All
3.21	Weapons such as knives, guns, and/or sharp objects?	None	Few	Some	Most	Almost all/All

[a] Item adapted for use in India.

Do not distribute without written consent. For more information, please contact:
Theresa S. Betancourt, Sc.D., M.A.
Director, Research Program on Children and Global Adversity | Harvard T.H. Chan School of Public Health
Department of Global Health and Population | Email: Theresa_Betancourt@harvard.edu

PART 4

The next set of questions ask about things relating to education for children (ages 0-18) at the [NAME OF SITE]. In addition, some questions also ask about economic security for children and their families. ***Please circle the answer to the following questions to the best of your knowledge.*** *You may not know all the answers. If you cannot answer a question, please leave the question blank.*

	**To the best of your knowledge, <u>how many of the children</u> (ages 0-18) [NAME OF SITE] currently:**	**None (0%)**	**Few (1%-25%)**	**Some (26%-50%)**	**Most (51%-75%)**	**Almost all/All (76%-100%)**
4.1	Attend school?	None	Few	Some	Most	Almost all/All
4.2	Of appropriate age attend an early education learning program, such as Aanganwadi Centers?[a]	None	Few	Some	Most	Almost all/All
4.3	Of appropriate age *attend* primary school?	None	Few	Some	Most	Almost all/All
4.4	Of appropriate age *complete* primary school?	None	Few	Some	Most	Almost all/All
4.5	Of appropriate age *attend* secondary school?	None	Few	Some	Most	Almost all/All
4.6	Of appropriate age *complete* secondary school?	None	Few	Some	Most	Almost all/All
4.7	Attend a non-formal and/or informal learning program such as skill building, vocational training, tailoring, IT courses, computer literacy, and/or informal apprenticeship programs?	None	Few	Some	Most	Almost all/All
4.8	Are on the street, at the market, at the railways, and/or at the worksite during school hours?	None	Few	Some	Most	Almost all/All
4.9	Are burned, hit, punched, shaken, kicked, and/or beaten by students, teachers, and/or other people in school?	None	Few	Some	Most	Almost all/All
4.10	Are inappropriately touched by students, teachers, and/or other people in school?	None	Few	Some	Most	Almost all/All
4.11	Are raped by students, teachers, and/or other people in school?	None	Few	Some	Most	Almost all/All
4.12	Are threatened, intimidated, yelled at harshly, name-called, accused, and/or humiliated by students, teachers, and/or other people in school?	None	Few	Some	Most	Almost all/All

[a] Item adapted for use in India.

Do not distribute without written consent. For more information, please contact:
Theresa S. Betancourt, Sc.D., M.A.
Director, Research Program on Children and Global Adversity | Harvard T.H. Chan School of Public Health
Department of Global Health and Population | Email: Theresa_Betancourt@harvard.edu

4.13	Are treated badly because of race, ethnicity, gender, caste, illness, disability, and/or religion by students, teachers, and/or other people?	None	Few	Some	Most	Almost all/All
4.14	Attend schools that have teachers who are frequently changing?	None	Few	Some	Most	Almost all/All
4.15	Attend schools that provide a mid-day meal?	None	Few	Some	Most	Almost all/All
4.16	Attend schools that employ teachers who have adequate years of schooling?	None	Few	Some	Most	Almost all/All
4.17	Have their own chair or bench to sit on at school?	None	Few	Some	Most	Almost all/All
4.18	Have their own textbooks at school?	None	Few	Some	Most	Almost all/All

	**To the best of your knowledge, how many of the children (ages 0-18) at the [NAME OF SITE] currently cannot attend school due to:**	**None (0%)**	**Few (1%-25%)**	**Some (26%-50%)**	**Most (51%-75%)**	**Almost all/All (76%-100%)**
4.19	Travel time and/or distance between [NAME OF SITE] and the school?					
4.20	School fees?	None	Few	Some	Most	Almost all/All
4.21	Need to earn money for household?	None	Few	Some	Most	Almost all/All
4.22	Transportation costs?	None	Few	Some	Most	Almost all/All
4.23	Cost of clothing such as uniforms and/or shoes?	None	Few	Some	Most	Almost all/All
4.24	Need to look after other children?	None	Few	Some	Most	Almost all/All
4.25	Need to look after an adult?	None	Few	Some	Most	Almost all/All
4.26	No schools in the area?	None	Few	Some	Most	Almost all/All
4.27	Not enough teachers?	None	Few	Some	Most	Almost all/All
4.28	Language spoken at school?	None	Few	Some	Most	Almost all/All
4.29	Lack of space in school for more students?	None	Few	Some	Most	Almost all/All
4.30	Pregnancy?	None	Few	Some	Most	Almost all/All
4.31	Marriage?	None	Few	Some	Most	Almost all/All

[a] Item adapted for use in India.

Do not distribute without written consent. For more information, please contact:
Theresa S. Betancourt, Sc.D., M.A.
Director, Research Program on Children and Global Adversity | Harvard T.H. Chan School of Public Health
Department of Global Health and Population | Email: Theresa_Betancourt@harvard.edu

	**To the best of your knowledge, how many of the children (ages 0-18) at the [NAME OF SITE] currently cannot attend school due to:**	**None (0%)**	**Few (1%-25%)**	**Some (26%-50%)**	**Most (51%-75%)**	**Almost all/All (76%-100%)**
4.32	Household chores within the family home?	None	Few	Some	Most	Almost all/All
4.33	Domestic labor such as cooking and/or cleaning outside the family home?	None	Few	Some	Most	Almost all/All
4.34	Selling things on the street?	None	Few	Some	Most	Almost all/All
4.35	Working in the fields?	None	Few	Some	Most	Almost all/All
4.36	Sex work such as prostitution?	None	Few	Some	Most	Almost all/All
4.37	Illegal activities such as selling drugs or stealing?	None	Few	Some	Most	Almost all/All
4.38	Scavenging for bottles and/or trash?	None	Few	Some	Most	Almost all/All
4.39	Hazardous work that harms health and/or safety?	None	Few	Some	Most	Almost all/All
4.40	Drug abuse?	None	Few	Some	Most	Almost all/All
4.41	Corporal punishment in schools?	None	Few	Some	Most	Almost all/All
4.42	Lack of proper documentation from previous schools?	None	Few	Some	Most	Almost all/All

PART 5

*The following questions ask about issues relating to income and economic stability for children (ages 0-18) and their families at the [NAME OF SITE]. **Please circle the answer to the following questions to the best of your knowledge.** You may not know all the answers. If you do not have **ANY** information to answer a question, please leave the question blank.*

	**To the best of your knowledge, in the last 6 months, how many of the children (ages 0-18) and/or families at the [NAME OF SITE] have been:**	**None (0%)**	**Few (1%-25%)**	**Some (26%-50%)**	**Most (51%-75%)**	**Almost all/All (76%-100%)**
5.1	Unable to afford basic needs, such as food, water, shelter, and/or clothing?	None	Few	Some	Most	Almost all/All
5.2	Unable to save money?	None	Few	Some	Most	Almost all/All

[a] Item adapted for use in India.

Do not distribute without written consent. For more information, please contact:
Theresa S. Betancourt, Sc.D., M.A.
Director, Research Program on Children and Global Adversity | Harvard T.H. Chan School of Public Health
Department of Global Health and Population | Email: Theresa_Betancourt@harvard.edu

5.3	Borrowing money from the bank, friends, and/or other individuals?	None	Few	Some	Most	Almost all/All
5.4	Owe money to others?	None	Few	Some	Most	Almost all/All
	**To the best of your knowledge, how many of the children (ages 0-18) and/or families at the [NAME OF SITE] have access to:**					
5.5	Vocational training opportunities?	None	Few	Some	Most	Almost all/All
5.6	Informal micro-lending schemes such as community loans?	None	Few	Some	Most	Almost all/All

[a] Item adapted for use in India.

Do not distribute without written consent. For more information, please contact:

Theresa S. Betancourt, Sc.D., M.A.
Director, Research Program on Children and Global Adversity | Harvard T.H. Chan School of Public Health
Department of Global Health and Population | Email: Theresa_Betancourt@harvard.edu
